# Supplementary material for: Content-rich biological network constructed by mining PubMed abstracts
Source: BMC Bioinformatics. 2004 Oct 8;5:147. doi: 10.1186/1471-2105-5-147 (PMC528731; doi:10.1186/1471-2105-5-147)
Supplement: Additional File 2 — The original results of the above study (non-essential files are deleted to keep the file size under the limit set by BMC bioinformatics). [file 1471-2105-5-147-S2.bz2 › chilibotAdditionalFile2/dip20/576ID9204896E10944/html/TEK_ANGPT2.html]

 


 **TEK** and **ANGPT2** 
  
Found 131 abstracts in PubMed, retrieved 20.  
 

 What does Google say? 
 PDF only 
| .edu only 

---

**Interactive relationship** (e.g. stimulation, inhibition, etc)

**Neutral relationship**- These data provide comprehensive evidence that VEGFR 1 and 2, and angiopoietin 1, angiopoietin 2  [ **ANGPT2** ]  and Tie 2  [ **TEK** ]  interactions may be involved in the preparation of endometrium for implantation, remodelling of the maternal vasculature and trophoblast invasion during the peri implantation period in this primate species.  Ref: 12887279 Reproduction, 2003
- Recent studies have implicated the Tie2  [ **TEK** ]  tyrosine kinase receptor and its main ligands angiopoietin 1 Ang 1 and angiopoietin 2  [ **ANGPT2** ]  Ang 2  [ **ANGPT2** ]  as crucial regulators of mural cell recruitment during angiogenesis.  Ref: 12876214 J Cell SciJ Cell Sci, 2003
- CONCLUSIONS VEGF and Ang2  [ **ANGPT2** ]  Tie2  [ **TEK** ]  interactions may play an important role in the pathogenesis of ROP.  Ref: 12815197 Ophthalmic Res, 2003
- The Tie2  [ **TEK** ]  receptor protein showed the same pattern as Ang 2  [ **ANGPT2** ] .  Ref: 12653688 Clin Sci Lond , 2003
- The change in capillary density paralleled with VEGF and Ang 2  [ **ANGPT2** ]  expression, but was inversely related to Tie2  [ **TEK** ]  phosphorylation.  Ref: 12649074 Am J Physiol Heart Circ Physiol< MedlineTA>Am J Physiol Heart Circ Physiol, 2003
- Angiopoietin 2  [ **ANGPT2** ]  Ang2  [ **ANGPT2** ]  appears to be a naturally occurring antagonist of the endothelial receptor tyrosine kinase Tie2  [ **TEK** ] , an important regulator of vascular stability.  Ref: 12692304 Proc Natl Acad Sci U S A, 2003
- Using a combination of immunohistochemistry, laser capture microdissection and real time quantitative reverse transcription PCR, we showed that tumor derived VEGF significantly up regulated the expression of Ang 2  [ **ANGPT2** ]  in host stroma endothelial cells, resulting in markedly increased Ang 2  [ **ANGPT2** ]  Tie 2  [ **TEK** ]  mRNA copy number ratio in vivo.  Ref: 12810677 Cancer Res, 2003

**Non-interactive relationship** (e.g. studied together, co-existance, homology, etc.)

- Cyclical mechanical stretch increased and Gö6976 or herbimycin A abrogated these increases the immunohistochemical labelling of angiopoietin 2  [ **ANGPT2** ]  and Tie2  [ **TEK** ]  receptor after a 6 h stretch.  Ref: 12653688 Clin Sci Lond , 2003
- These included familiar endothelial cell associated genes such as VEGF, VEGFR 1, VEGFR 2, angiopoietin 2  [ **ANGPT2** ] , Tie1, Tie2  [ **TEK** ] , Edg1 receptor, VE cadherin, claudin 5, connexin 37, CD31, and CD34.  Ref: 12842817 Am J Physiol Heart Circ Physiol< MedlineTA>Am J Physiol Heart Circ Physiol, 2003
- Here, we studied the roles of Ang1, its natural antagonist Ang2  [ **ANGPT2** ] , and their receptor Tie2  [ **TEK** ]  in rat cardiac allograft arteriosclerosis.  Ref: 12628953 Circulation, 2003
- Angiopoetin 1 Ang1, angiopoietin 2  [ **ANGPT2** ]  Ang2  [ **ANGPT2** ] , Tie 1, and Tie 2  [ **TEK** ]  were measured immediately after relief of occlusion, 60 min, 4, and 72 h after reperfusion by Northern blot, Western blot, and immunohistochemical staining.  Ref: 12737621 Clin Sci Lond < MedlineTA>Clin Sci Lond , 2003
- mRNAs for VEGF, VEGFR 1 and VEGFR 2, angiopoietin 1, angiopoietin 2  [ **ANGPT2** ]  and their receptor Tie 2  [ **TEK** ]  were localized and quantified by in situ hybridization.  Ref: 12887279 Reproduction, 2003
- Immunohistochemical study also showed increased immunoreactivity of Ang2  [ **ANGPT2** ]  and Tie2  [ **TEK** ]  in the jeopardized myocardium after ischemia reperfusion.  Ref: 12737621 Clin Sci Lond < MedlineTA>Clin Sci Lond , 2003
- Similar to protein expression, the levels of angiopoietin 2  [ **ANGPT2** ]  and Tie2  [ **TEK** ]  receptor mRNAs in HUVECs increased 3.1 fold and 2.5 fold respectively after stretch for 6 h.  Ref: 12653688 Clin Sci Lond , 2003
- In conclusion, our data suggest that both hypoxia and VEGF treatment differentially regulate the angiopoietin Tie2  [ **TEK** ]  system in the two vascular cells and that, particularly in BRP, the regulation of Ang1, but not Ang2  [ **ANGPT2** ] , and Tie2  [ **TEK** ]  expression may play an important role in vascular development.  Ref: 12686170 Microvasc Res, 2003
- Using RT PCR and immunohistochemistry we demonstrate that testicular vascular endothelial growth factor A VEGF A, ang 1, ang 2  [ **ANGPT2** ]  and the tie 2  [ **TEK** ]  angiopoietin receptor are expressed in the testis.  Ref: 12773423 Biol Reprod< MedlineTA>Biol Reprod, 2003
- In conclusion, Ang2  [ **ANGPT2** ]  and Tie2  [ **TEK** ]  expressions increased after ischemia reperfusion in the rat ventricular myocardium, while Ang1 and Tie1 expressions did not.  Ref: 12737621 Clin Sci Lond < MedlineTA>Clin Sci Lond , 2003
- These increases in angiopoietin 2  [ **ANGPT2** ]  and Tie2  [ **TEK** ]  receptor proteins at 6 h were blocked by the addition 30 min before stretch of the protein kinase C inhibitor Gö6976 16 nM or the tyrosine kinase inhibitor herbimycin A 24 microM.  Ref: 12653688 Clin Sci Lond , 2003
- The level of Ang 1 and Tie 2  [ **TEK** ]  mRNAs, but not that of Ang 2  [ **ANGPT2** ]  mRNA gradually decreased during a 14 d incubation of cord blood CD34 cells in a liquid culture.  Ref: 12605658 Eur J Haematol, 2003
- Immunoprecipitation showed phosphorylation of MPC Tie2  [ **TEK** ]  on tyrosine residues upon stimulation with Ang 1 or Ang 2  [ **ANGPT2** ] .  Ref: 12876214 J Cell SciJ Cell Sci, 2003
- To test this hypothesis we measured capillary density, and the expressions of VEGF, angiopoietin 1 and 2 Ang 1 and Ang 2  [ **ANGPT2** ] , respectively and the Tie 2  [ **TEK** ]  receptor and its phosphorylation state pY Tie2  [ **TEK** ]  during repetitive episodes of myocardial ischemia in chronically instrumented canines.  Ref: 12649074 Am J Physiol Heart Circ Physiol< MedlineTA>Am J Physiol Heart Circ Physiol, 2003
- ... Ang 2  [ **ANGPT2** ]  and Ang 3 inhibit Ang 1 induced Tie2  [ **TEK** ]  phosphorylation.  Ref: 12783598 Expert Opin Investig Drugs, 2003
- To investigate the specific role of Ang2  [ **ANGPT2** ]  in the adult vasculature, we generated a nuclease resistant RNA aptamer that binds and inhibits Ang2  [ **ANGPT2** ]  but not the related Tie2  [ **TEK** ]  agonist, angiopoietin 1.  Ref: 12692304 Proc Natl Acad Sci U S A, 2003
- RESULTS A higher expression of Ang 1, Ang 2  [ **ANGPT2** ] , and Tie 2  [ **TEK** ]  mRNAs was detected in CD34 CD133 cord blood cells as compared with CD34 CD133 fraction, but no expression of these genes was detected in burst forming unit erythroid BFU E nor colony forming unit granulocyte macrophage CFU GM colonies.  Ref: 12605658 Eur J Haematol, 2003
- CONCLUSIONS A specific increase of Ang 1 Ang 2  [ **ANGPT2** ]  ratio in FNH, in the presence of the functional Tie 2  [ **TEK** ]  receptor, might be involved in the formation of hyperplastic and dystrophic vessels of FNH.  Ref: 12612904 Gastroenterology, 2003
- Tie 2  [ **TEK** ]  messenger RNA, the receptor of Ang 1 and Ang 2  [ **ANGPT2** ] , was detected at the same level in FNH as in normal liver.  Ref: 12612904 Gastroenterology, 2003
- Polyclonal antibodies specific for Ang1, Ang2  [ **ANGPT2** ] , Tie2  [ **TEK** ]  and VEGF were used for immunostaining.  Ref: 12815197 Ophthalmic Res, 2003
- MATERIAL AND METHODS We have measured the expression of angiogenesis regulating genes angiopoietin 1 Ang 1, angiopoietin 1 Ang 2  [ **ANGPT2** ]  and their receptor Tie 2  [ **TEK** ] , vascular endothelial growth factor VEGF and its receptors VEGFR 1 and VEGFR 2 in sorted population of CD34 and CD34 CD133 cells from human cord blood and bone marrow, and in their differentiating progeny, using real time reverse transcriptase polymerase chain reaction.  Ref: 12605658 Eur J Haematol, 2003
- In this study, we investigated the expression of Ang 1, Ang 2  [ **ANGPT2** ] , Tie 2  [ **TEK** ] , and vascular endothelial growth factor VEGF in surgically resected specimens from 46 patients with HCC to determine their potential role in tumor angiogenesis and its progression.  Ref: 12717391 Hepatology, 2003
- Ang2  [ **ANGPT2** ]  and Tie2  [ **TEK** ]  immunoreactivity was induced mainly in capillaries and postcapillary venules in chronic allografts when compared with syngeneic controls, but no immunoreactivity was found in arterial endothelium.  Ref: 12628953 Circulation, 2003
- The study of angiopoietin 1 Ang 1 and angiopoietin 2  [ **ANGPT2** ]  Ang 2  [ **ANGPT2** ] , ligands of receptor tyrosine kinase 2 Tie 2  [ **TEK** ] , showed that while stroma derived Ang 2  [ **ANGPT2** ]  was increased, epidermal Ang 1 expression was completely abolished at early papilloma formation.  Ref: 12766907 Mol Carcinog, 2003
- Ang2  [ **ANGPT2** ]  stained positively in 18 of 38 47.3% serial sections where Tie2  [ **TEK** ]  was present, and was also co expressed with VEGF and factor VIII.  Ref: 12815197 Ophthalmic Res, 2003
- However, using bovine aortic endothelial cells BAEC, we found that only Ang2  [ **ANGPT2** ]  expression, but neither Ang1 nor Tie2  [ **TEK** ] , responded to these two angiogenic stimuli, which was consistent with many previous reports.  Ref: 12686170 Microvasc Res, 2003
- Thus cyclical mechanical stretch activates the expression of angiopoietin 2  [ **ANGPT2** ]  and the Tie2  [ **TEK** ]  receptor, but not angiopoietin 1 or the Tie1 receptor, in cultured HUVECs.  Ref: 12653688 Clin Sci Lond , 2003
- Cyclical mechanical stretch enhances angiopoietin 2  [ **ANGPT2** ]  and Tie2  [ **TEK** ]  receptor expression in cultured human umbilical vein endothelial cells.  Ref: 12653688 Clin Sci Lond , 2003
- Colocalization of tie2  [ **TEK** ] , angiopoietin 2  [ **ANGPT2** ]  and vascular endothelial growth factor in fibrovascular membrane from patients with retinopathy of prematurity.  Ref: 12815197 Ophthalmic Res, 2003
- Increased expression of angiopoietin 2  [ **ANGPT2** ]  and Tie2  [ **TEK** ]  receptor in a rat model of myocardial ischemia reperfusion.  Ref: 12737621 Clin Sci Lond < MedlineTA>Clin Sci Lond , 2003
